# The pioneer factor activity of c-Myb involves recruitment of p300 and induction of histone acetylation followed by acetylation-induced chromatin dissociation

**DOI:** 10.1186/s13072-018-0208-y

**Published:** 2018-06-28

**Authors:** Bettina M. Fuglerud, Marit Ledsaak, Marie Rogne, Ragnhild Eskeland, Odd S. Gabrielsen

**Affiliations:** 1 Department of Biosciences, University of Oslo, P.O. Box 1066, 0316 Blindern, Oslo, Norway; 2 Centre for Cancer Cell Reprogramming, Institute of Clinical Medicine, Faculty of Medicine, University of Oslo, Oslo, 0379 Norway

## Abstract

**Background:**

The concept of pioneer transcription factors is emerging as an essential part of the epigenetic regulation, taking place during cell development and differentiation. However, the precise molecular mechanism underlying pioneer factor activity remains poorly understood. We recently reported that the transcription factor c-Myb acts as a pioneer factor in haematopoiesis, and a point mutation in its DNA binding domain, D152V, is able to abrogate this function.

**Results:**

Here, we show that specific histone modifications, including H3K27ac, prevent binding of c-Myb to histone tails, representing a novel effect of histone modifications, namely restricting binding of a pioneer factor to chromatin. Furthermore, we have taken advantage of the pioneer-defect D152V mutant to investigate mechanisms of c-Myb’s pioneer factor activity. We show that c-Myb-dependent transcriptional activation of a gene in inaccessible chromatin relies on c-Myb binding to histones, as well as on c-Myb interacting with the histone acetyltransferase p300. ChIP assays show that both wild type and the D152V mutant of c-Myb bind to a selected target gene at its promoter and enhancer, but only wild-type c-Myb causes opening and activation of the locus. Enhancement of histone acetylation restores activation of the same gene in the absence of c-Myb, suggesting that facilitating histone acetylation is a crucial part of the pioneer factor function of c-Myb.

**Conclusions:**

We suggest a pioneer factor model in which c-Myb binds to regions of closed chromatin and then recruits histone acetyltransferases. By binding to histones, c-Myb facilitates histone acetylation, acting as a cofactor for p300 at c-Myb bound sites. The resulting H3K27ac leads to chromatin opening and detachment of c-Myb from the acetylated chromatin. We propose that the latter phenomenon, acetylation-induced chromatin dissociation, represents a mechanism for controlling the dynamics of pioneer factor binding to chromatin.

**Electronic supplementary material:**

The online version of this article (10.1186/s13072-018-0208-y) contains supplementary material, which is available to authorized users.

## Background

In eukaryotes, nucleosomes provide steric constraints on how transcription factors can bind DNA. Some transcription factors have the ability to associate with condensed chromatin independently of other factors and modulate chromatin structure. These factors are termed pioneer transcription factors and are the first factors to access a regulatory region. Upon binding to chromatin, pioneer factors increase the accessibility of their target site and thereby allow access to other transcription factors and chromatin modifiers [1, 2]. A large number of identified pioneer factors are emerging, but the precise molecular mechanism underlying pioneer factor function is still an unresolved question in the field. The exception is FoxA1, which has emerged as the prototype pioneer factor. The DNA binding domain of FoxA1 resembles that of linker histones, which makes this pioneer factor structurally suited to bind to nucleosomes, thus facilitating chromatin opening [3, 4]. However, this seems to be a special case for FoxA1 and we have limited understanding of the mechanistic details of other pioneer factors.

Pioneer factors are commonly found bound at enhancers, where chromatin accessibility is highly variable and cell-type specific [1, 5]. Specific histone post-translational modifications (PTMs) are associated with enhancers and their state of activity. For example, active enhancers are acetylated at histone 3 lysine 27 (H3K27ac) and mono-methylated at histone 3 lysine 4 (H3K4me1), generated by histone acetyltransferases (HATs) and histone methyltransferases (HMTs), respectively [6]. H3K4me1 marks both poised and active enhancers, whereas H3K27ac is found at active enhancers and is acquired almost exclusively in the context of pre-existing H3K4me1 at enhancers [7]. Most pioneer factors interact with chromatin modifiers, and in some studies they have been shown to facilitate H3K27ac at enhancers [8–10]. The consequences of enhancer histone acetylation include binding by proteins containing bromodomains, long-distance enhancer–promoter communication by chromatin looping, as well as formation of a more relaxed chromatin structure resulting from the removal of positive charge from histones [6, 11, 12].

We recently reported that the transcription factor c-Myb functions as a pioneer factor in the regulation of genes essential for haematopoiesis [13]. Furthermore, we identified a pioneer-defect mutant of c-Myb, aspartate 152 to valine (D152V), which has a weakened interaction with H3 while its DNA binding is intact [13]. c-Myb has previously been shown to recruit the HAT CBP to the *TAL1* oncogene, resulting in extensive H3K27ac and the generation of an oncogenic super-enhancer [10]. The interaction between c-Myb and p300 has also been shown to be required for the induction of acute myeloid leukaemia (AML) and seems to be a promising therapeutic target for AML treatment [14, 15]. This suggests that c-Myb acts in concert with p300/CBP to activate enhancers for defining cell identity during normal haematopoiesis, which in turn may aberrantly activate expression of oncogenes during development of leukaemia. Moreover, it suggests that the pioneer factor mechanism of c-Myb might involve the facilitation of H3K27ac at enhancers by p300/CBP. In our study of the D152V mutant of c-Myb, we discovered that the genes that this mutant fails to regulate in a myeloid cell line (K562) are involved in AML signalling, strongly suggesting that the pioneer function of c-Myb is directly connected to its functions in AML. The D152V mutant is an important asset in the study of the pioneer factor functions of c-Myb and may help to identify specific pioneer factor mechanisms, which might also be applicable to other pioneer factors operating in other cell types.

In the present work, we have studied the effect of histone acetylation on c-Myb-dependent activation of a chromatin-embedded gene and how the binding of c-Myb to histones is affected by acetylation. We reveal that histone modifications result in impaired binding of c-Myb to histones. In particular, c-Myb was affected by modifications of the H3 tail (H3R26 citrullination, H3K27 acetylation and H3S28 phosphorylation). At one of the pioneer factor target genes of c-Myb, *mim*-*1*, the recruitment of p300 and histone acetylation seems to be essential for activation. Based on these observations, we propose a specific mechanism of action of c-Myb pioneer factor function, where c-Myb recruits p300/CBP to closed regions of chromatin and facilitates histone acetylation. This suggests that for c-Myb to fully function as a pioneer factor, the interaction with both H3 and p300/CBP needs to be intact. Furthermore, we propose that histone acetylation at specific sites cause dissociation of c-Myb from chromatin upon activation of target genes. The latter is a novel consequence of histone acetylation in regulation of pioneer factor binding to chromatin.

## Results

### Binding of c-Myb to native histones from K562 cells is strongly impaired by histone acetylation

The c-Myb mutant D152V contains an amino acid substitution of valine for aspartate within its DNA binding domain (DBD). This mutation results in a weakened interaction with H3, but the binding to DNA is not affected [13]. The mutated aspartate residue is located to an acidic patch in the c-Myb DBD and results in reduced negative charge. c-Myb contains three of these acidic patches, one in each of the repeats constituting the DBD, named R1, R2 and R3, and these have previously been suggested to bind to histone tails [13]. Considering the basic nature of histone tails, it was expected that the loss of negative charge caused by D152V in the c-Myb DBD would result in a weakened histone binding. We therefore asked the question whether the opposite might also be the case; namely a weakened c-Myb–histone interaction due to loss of positive charge in histones. Acetylation neutralizes the positive charge of the lysine residues on histones, and therefore, we tested the effect of chromatin acetylation on c-Myb binding to histones.

**Fig. 1:**
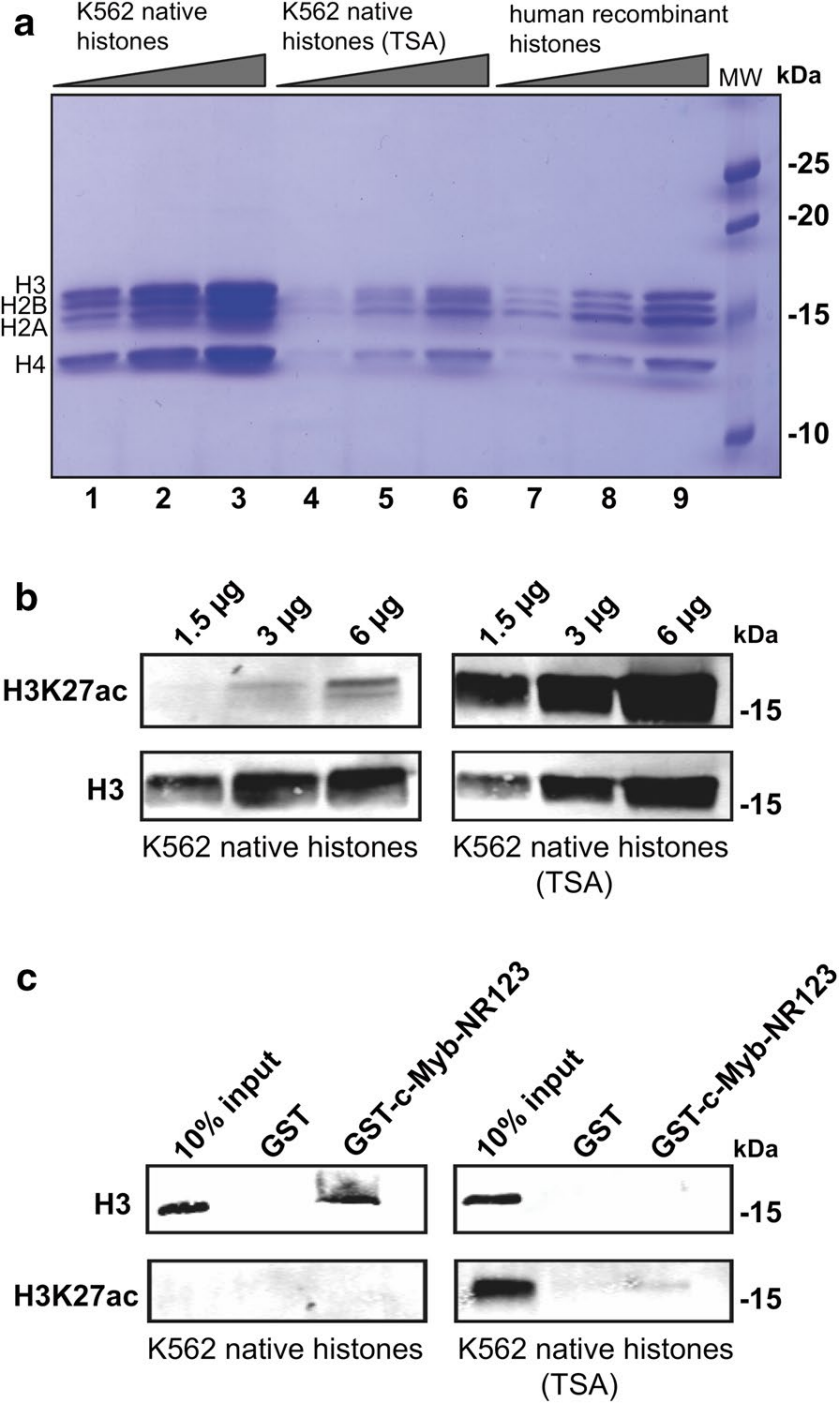
Histone acetylation prevents c-Myb binding to native K562 histones. **a** 18% SDS-PAGE of native histones isolated from K562 cells treated with DMSO (lane 1–3) or TSA (lane 4–6) and recombinant human histones loaded as reference (lane 7–9). **b** Acetylation of K562 histones was validated by western blotting using anti-H3K27ac primary antibody (ab177178, Abcam) and anti-H3 primary antibody (ab1791, Abcam) as reference. **c** GST pull-down assay performed with GST-fused c-Myb DBD (NR123) and 6 µg native K562 histone octamers. The western blot was analysed by using anti-H3 and anti-H3K27ac primary antibodies. About 10% (0.6 µg) of the K562 histones was loaded as reference

We treated K562 cells with Trichostatin A (TSA) to inhibit histone deacetylase (HDAC) activity and increase histone acetylation levels in the cells. Native histone octamers from K562 cells treated with TSA or DMSO (control) were purified, and the increased acetylation was confirmed by western blotting using H3K27ac antibody (Fig. 1a, b). The purified octamers were used in a GST pull-down assay with the DBD of c-Myb fused to GST as bait. c-Myb bound to H3 from the native octamers not treated with TSA, but the binding was lost in the assay with acetylated histone octamers (Fig. 1c). Thus, removal of negative charge in c-Myb or positive charge in histones will result in a weakened interaction between c-Myb and histone H3.

### Specific histone modifications prevent c-Myb binding to histone tails

**Fig. 2:**
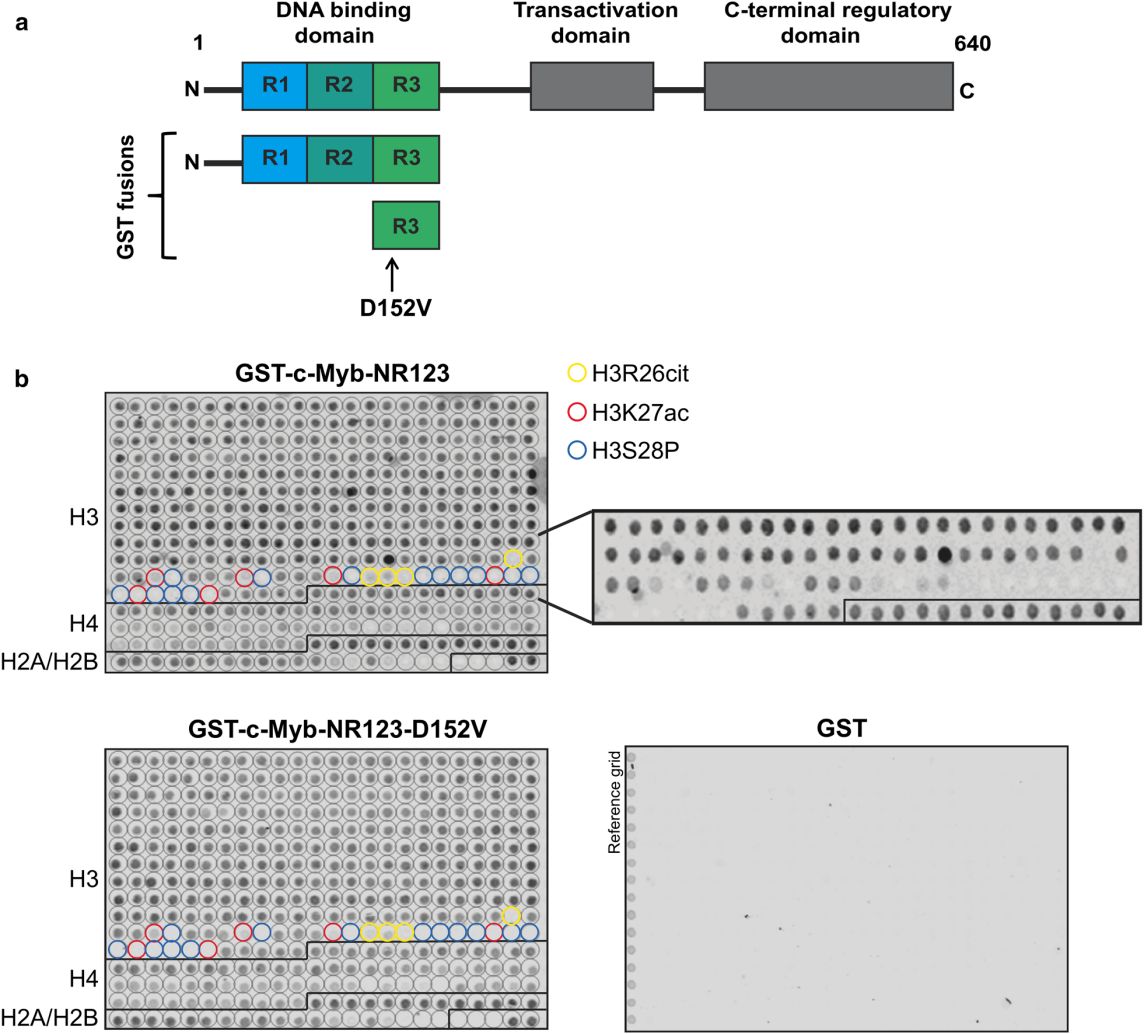
c-Myb histone binding is impaired by specific histone modifications. **a** Schematic figure of the full-length c-Myb protein and the regions fused to GST in GST pull-down assays and peptide binding assays. The D152V mutation is indicated with an arrow. **b** Peptide arrays containing 384 different histone tail modification combinations incubated with GST, GST-c-Myb-NR123 or GST-c-Myb-NR123-D152V and detected with anti-GST primary antibody (A00865, GenScript). The locations of H3R26 citrullination, acetylation and H3S28 phosphorylation (alone and in combinations with other modifications) are indicated with yellow, red and blue circles, respectively. An enlarged version of the region containing these modifications for the array incubated with GST-c-Myb-NR123 without annotations is also shown

Histone acetylation resulted in a weakened binding of c-Myb to histones. However, TSA treatment is unspecific and we could not know whether c-Myb binding is more sensitive to acetylation of some residues than others. To determine which histone modifications prevent c-Myb binding, we used a histone tail peptide array containing 59 acetylation, methylation, phosphorylation and citrullination (also known as deimination) modifications on the N-terminal tails of histones H2A, H2B, H3 and H4 in 384 different combinations (including unmodified histone tails) in duplicate. In two independent experiments, we observed that the DBD of c-Myb fused to GST bound to most of the peptides on the array, including the unmodified histone tails (Fig. 2). Interestingly, a few specific histone modifications seem to prevent c-Myb binding (Fig. 2b, middle panel). We observed the most specific effect on the H3 tail, where citrullination of R26 (H3R26cit), acetylation of K27 (H3K27ac) and phosphorylation of S28 (H3S28P) prevented c-Myb binding, both alone and in combinations with other modifications. We also observed weakened binding to acetylated and phosphorylated H4 and H2B, but only when multiple residues were acetylated or phosphorylated, and thus, the effect was less specific than for H3.

The three specific modifications that prevented c-Myb from binding to the H3 tail are all associated with chromatin decompaction and activation of transcription [16–18]. Moreover, these modifications result in less positive charge of histones tails, either by the neutralization of a positive charge, which is the case for H3R26cit and H3K27ac, or by introduction of a negative charge, as in the case of H3S28P. This may explain the loss of c-Myb binding, since the interaction between c-Myb and histones is likely to be mediated through electrostatic interactions between the acidic patches of the c-Myb DBD and the positively charged histone tails. Of note, methylation of H3R26 and H3K27 did not affect c-Myb binding, possibly because histone methylation does not alter the charge of the histone tail.

We also incubated a histone peptide array with the DBD of c-Myb D152V fused to GST and observed the same binding pattern as for wild-type c-Myb (Fig. 2b, lower panel). This mutation is located to the R3 repeat of the DBD and results in a loss of H3 binding in the R3 (Fig. 2a). The R2 repeat also contains a histone binding site, which is able to bind to H3 even though R3 is mutated, resulting in a weakened binding of the full DBD to H3, but not complete loss [13]. To test whether R3 alone has the same histone binding pattern as the full DBD, we incubated histone peptide arrays with wild-type and mutant R3 fused to GST. We observed the same binding pattern for wild-type R3 as the full DBD, but R3 D152V did not bind to any of the peptides on the array (Additional file 1: Figure S1). This suggest that the histone binding sites in the c-Myb DBD binds to peptides from all four canonical histones and that binding to all four histones is weakened by the D152V mutation.

Taken together, the binding of c-Myb to histone tails seems to be quite unspecific, as c-Myb binds to the tails of all four canonical histones, regardless of modifications. However, the modifications that prevent c-Myb from binding histone tails are very specific. In particular, modifications of the residues 26–28 of H3 causing less positive charge of the H3 tail result in a loss of c-Myb binding. Many acetylations elsewhere in H3 that is also reducing the charge of the histone tail had no effect on c-Myb binding. This may reflect a preferred positioning of c-Myb binding to this region of the histone tail.

### Histone acetylation can restore activation of a chromatin-embedded gene

Due to the specific effect of H3K27ac on c-Myb histone binding, we asked how histone acetylation affects c-Myb-dependent transcriptional activation and in particular, how the transcriptional activation of a pioneer target gene of c-Myb is affected. The chicken *mim*-*1* gene is one of the most thoroughly studied c-Myb target genes and a well-established model system to study the initial events of chromatin opening and how c-Myb activates its target genes [19]. *mim*-*1* is expressed in myelomonocytic cells, and the expression only occurs in the presence of c-Myb and is associated with chromatin opening at the *mim*-*1* enhancer and promoter [20, 21]. We recently reported that the c-Myb D152V mutant is unable to activate transcription of endogenous *mim*-*1* in the chicken macrophage cell line HD-11, while it was fully able to activate the same promoter on a transfected plasmid, suggesting that the D152V mutation specifically interferes with the ability of c-Myb to activate transcription in a chromatin context [13].

**Fig. 3:**
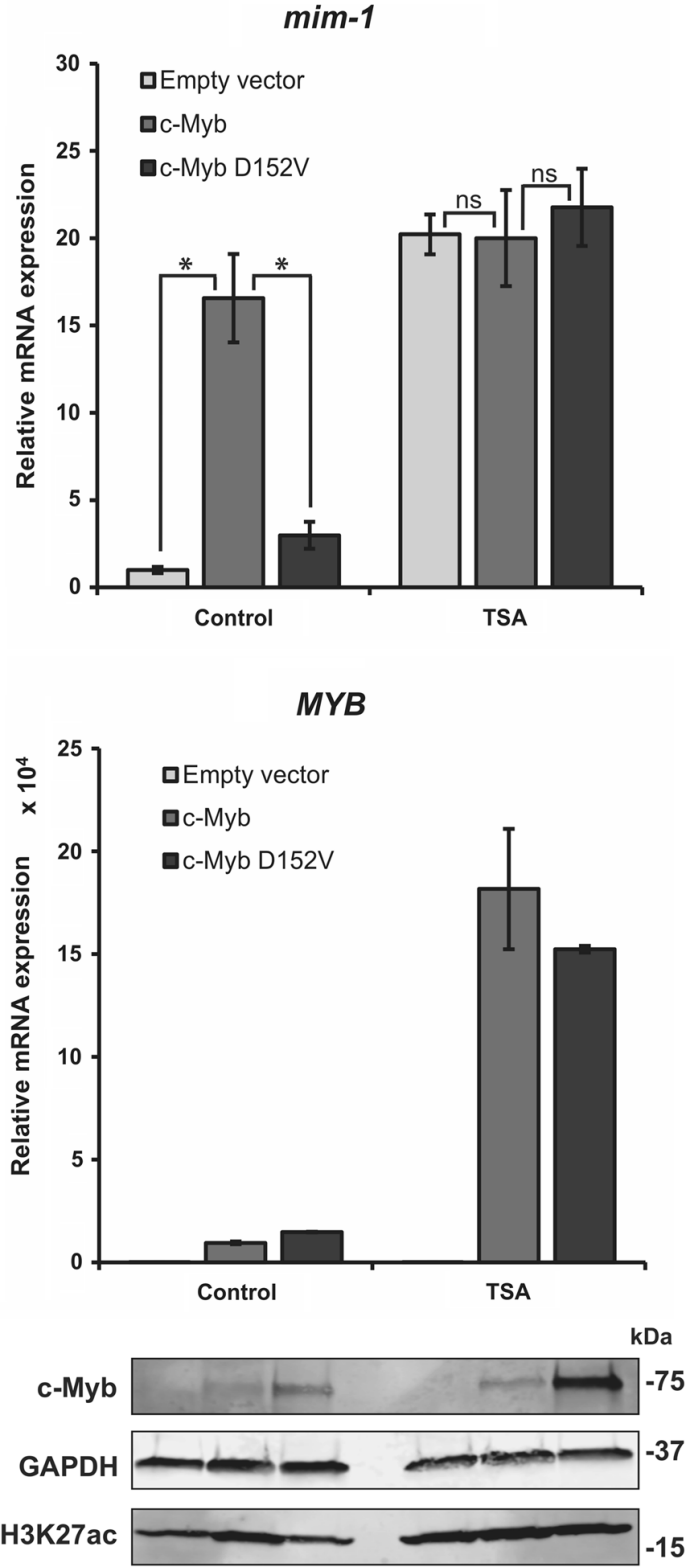
c-Myb-dependent transcriptional activation of a chromatin-embedded gene involves increased histone acetylation. HD-11 cells were transfected with plasmids encoding full-length HA-tagged c-Myb or c-Myb D152V. The cells were treated with 15 ng/ml TSA or DMSO 8 h after transfection. Total RNA was isolated 24 h after transfection, and *mim*-*1* and *MYB* expression was measured by qRT-PCR. The values of RNA expression were normalized to the relative amount of the reference gene *hprt*. The western blots were analysed using anti-c-Myb (ab45150, Abcam), anti-GAPDH (AM4300, Invitrogen) and anti-H3K27ac primary antibodies. The qRT-PCR results are presented as mean ± SEM of three independent biological replicates. Significance was evaluated by unpaired, two-tailed *t* tests on selected pairs and indicated with *p* values (*p < 0.05; ns *p* > 0.05). No *p* values were calculated for the MYB expression since only two biological replicates were used for this measurement

To determine the role of histone acetylation in c-Myb-dependent transcriptional activation, we treated HD-11 cells with TSA to increase global acetylation levels in the cells and analysed the level of *mim*-*1* expression in cells transfected with wild-type c-Myb or c-Myb D152V. In the cells not treated with TSA, wild-type c-Myb activated transcription of endogenous *mim*-*1* as expected, whereas D152V failed to do so (Fig. 3, upper left). TSA treatment resulted in high expression of *mim*-*1* also in the cells transfected with empty vector and c-Myb D152V, indicating that acetylation can compensate for the defect of D152V. Interestingly, the level of *mim*-*1* expression in the cells transfected with wild-type c-Myb was not affected by the TSA treatment (Fig. 3, upper right). The latter strongly suggests that c-Myb itself cause increased histone acetylation at the *mim*-*1* locus and acetylation levels will not further increase upon TSA treatment. A similar profile was observed for another c-Myb responsive gene, the *lysozyme* (*LYZ*) gene (Additional file 2: Figure S2). The expression level of *MYB* is highly increased by the TSA treatment (Fig. 3, lower panel), perhaps due to increased expression of factors activating *MYB* expression caused by the higher level of histone acetylation upon TSA treatment. However, this does not seem to be the reason for the rescue of *mim*-*1* expression, as *mim*-*1* expression is increased by TSA treatment even in the cells not transfected with c-Myb. Of note, the D152V mutant seems to be highly stable in HD-11 cells, as judged from its strong western blot signal. We previously reported that this is the case in specific cell types [13]. One possible explanation for this phenomenon may be that due to its inability to cause increased histone acetylation, c-Myb D152V will have a longer residence time bound to chromatin because histone acetylation facilitates detachment of c-Myb from chromatin. Assuming that c-Myb is degraded in the cell after having performed its pioneer factor function and dissociated from chromatin, c-Myb D152V would be present in higher protein levels because of its slower dynamics. We tested whether this was reflected in differential chromatin association upon salt extraction of nuclei, but did not observe any significant change in the elution profile of the mutant versus the wild type (Additional file 3: Figure S3). This phenomenon of lower stability of an active version of a factor has been observed for several transcription factors and appears to allow tight control of transcription by ensuring that activation of a gene is connected to the levels of the regulators of that gene [22].

### Histone binding and p300 recruitment is required for c-Myb to fully activate transcription of a chromatin-embedded gene

c-Myb has been found to recruit p300/CBP to target genes and facilitate histone acetylation [10, 23]. Since it is the histone binding that is weakened in the D152V mutant, we next asked whether a mutant in the p300 interaction region of c-Myb would be able to activate transcription of *mim*-*1*. The c-Myb mutant M303V was identified by mice mutagenesis screening and the mice carrying this mutation had elevated levels of megakaryocytes and platelet production, as well as blocks in T cell, B cell and red blood cell development [24]. Of note, this phenotype is strikingly similar to the phenotype of mice harbouring the D152V mutation of c-Myb [25]. The M303V mutation is located to the transactivation domain of c-Myb, which mediates the interaction with p300 [26], and was reported to have weakened interaction with p300 resulting in inefficient recruitment of p300 to target genes [24].

**Fig. 4:**
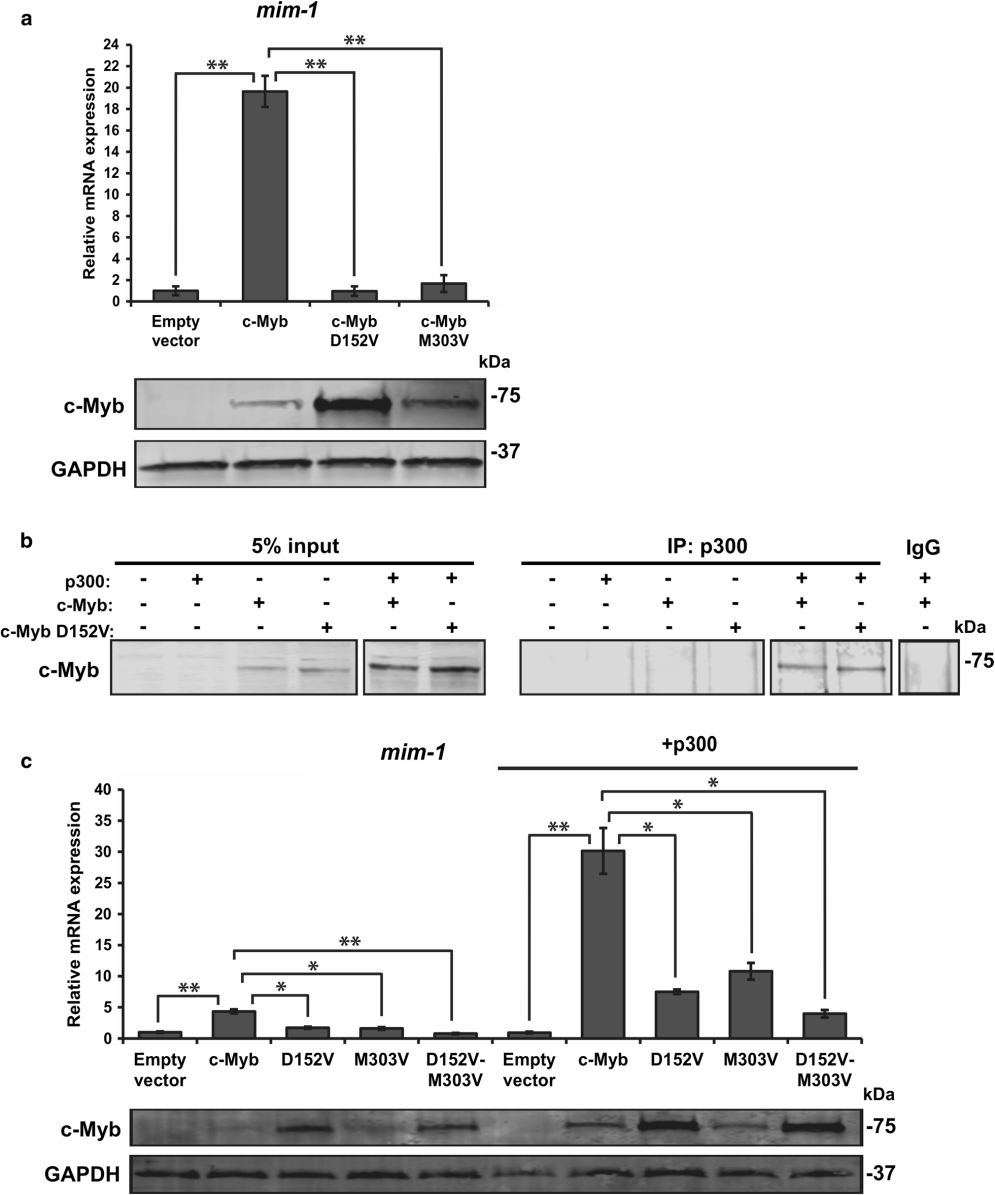
Interaction with both H3 and p300 is essential for c-Myb-dependent activation of a chromatin-embedded gene. **a** HD-11 cells were transfected with plasmids encoding full-length HA-tagged c-Myb, c-Myb D152V and c-Myb M303V. Total RNA was isolated 24 h after transfection, and *mim*-*1* expression was measured by qRT-PCR relative to the reference gene *hprt*. The western blots were analysed using anti-c-Myb and anti-GAPDH primary antibodies. **b** Co-immunoprecipitation was performed with COS-1 cells transfected with plasmids encoding full-length HA-tagged c-Myb, c-Myb D152V and p300-Myc and lysed 24 h after transfection. Immunoprecipitation was performed using anti-p300 antibody (SC-585, Santa Cruz Biotechnology), and the proteins were detected by western blotting using anti-HA primary antibody (H6908, Sigma-Aldrich). 5% of total transfected cell lysate was loaded as reference. **c** HD-11 cells were transfected with plasmids encoding full-length HA-tagged c-Myb, c-Myb D152V, c-Myb M303V, c-Myb D152V-M303V and p300-Myc. Total RNA was isolated 24 h after transfection, and *mim*-*1* expression was measured by qRT-PCR relative to the reference gene *hprt*. The western blots were analysed using anti-HA and anti-GAPDH primary antibodies. The qRT-PCR results in **a** and **c** are presented as mean ± SEM of three independent biological replicates. Significance was evaluated by unpaired, two-tailed *t* tests on selected pairs and indicated with *p* values (*p < 0.05; **p < 0.01; ns *p* > 0.05)

We transfected HD-11 cells with wild-type c-Myb, c-Myb D152V and M303V and analysed the expression level of *mim*-*1*. We observed the same defect of c-Myb M303V in activating transcription of endogenous *mim*-*1* as c-Myb D152V, suggesting that both histone binding and p300 involvement are crucial for activation of the *mim*-*1* gene in HD-11 cells (Fig. 4a). To be certain that the D152V mutation does not disrupt the interaction with p300, we performed a co-immunoprecipitation experiment and observed that both wild-type c-Myb and D152V are immunoprecipitated together with p300 (Fig. 4b). p300 acts as a co-activator of c-Myb transcriptional activity, and since the interaction with both histones and p300 seem to be necessary for c-Myb to activate transcription of *mim*-*1*, and possibly for the induction of histone acetylation, we decided to test whether an increased supply of p300 might have an effect on the two c-Myb mutants. We transfected HD-11 cells with the c-Myb constructs in the presence or absence of co-transfected p300 and analysed the *mim*-*1* expression levels. We also included a D152V-M303V double mutant of c-Myb. As expected, we observed an enhancement of *mim*-*1* expression in cells co-transfected with c-Myb and p300 (Fig. 4c). Cells co-transfected with p300 and either c-Myb D152V or M303V had enhanced expression of *mim*-*1* compared to the cells without ectopic p300 expression, but not as high as for wild-type c-Myb. The cells transfected with the double mutant had even lower expression of *mim*-*1* than those transfected with c-Myb D152V or c-Myb M303V when co-transfected with p300, but the *mim*-*1* expression was enhanced compared to the cells without ectopic p300 expression. This suggests that even though both histone interaction and p300 involvement need to be intact for c-Myb to fully activate transcription of a chromatin-embedded gene, an excess supply of p300 is able to partially compensate for the defects of mutants where these interactions are weakened.

### ChIP analysis of the *mim*-*1* locus in HD-11 cells

**Fig. 5:**
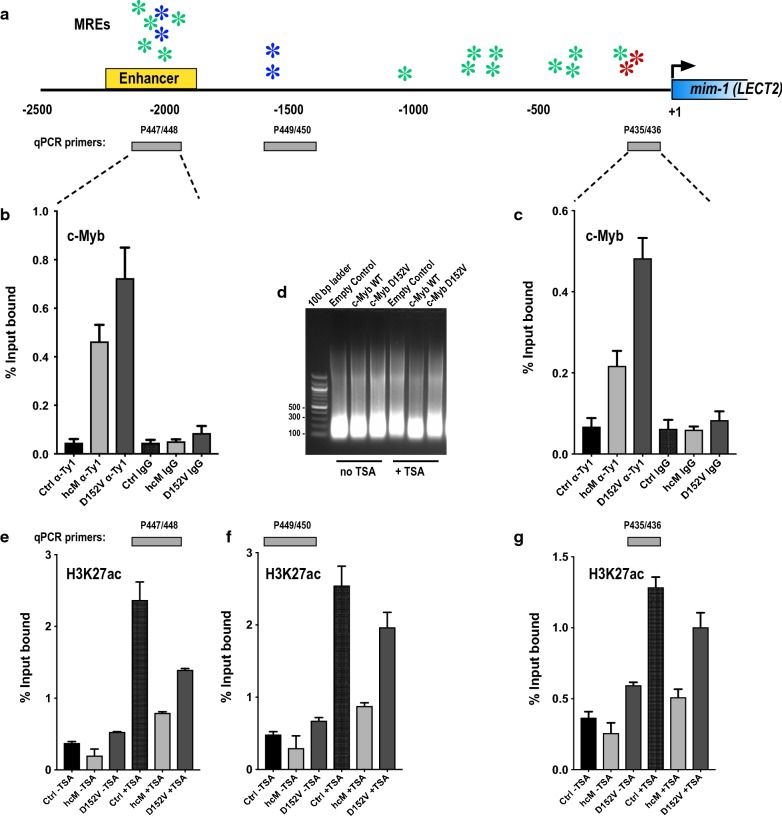
ChIP analysis of c-Myb occupancy and H3K27ac levels at the *mim*-*1* locus. **a** Map of the *mim*-*1* locus (*LECT2* leucocyte cell derived chemotaxin 2 (*Gallus gallus (Chicken)*). The enhancer mapped in [28] is indicated. Myb recognition elements (MREs) are indicated by colour-coded asterisk (red: the strong MRE = YAACGG [27], green: MRE = YAACNG and blue: double MREs with palindromic orientation). The amplicons used for qRT-PCR quantification are indicated. **b**, **c** Occupancy of c-Myb wild-type (hcM) and D152V as measured by ChIP and qRT-PCR at the indicated regions. IgG IP controls are included. Bar graphs represent the mean ± SEM of three independent biological replicates each measured in triplicate. **d** Fragmentation of chromosomal DNA of one replicate. **e**–**g** Levels of H3K27ac as measured by ChIP and qRT-PCR at the indicated regions. Bar graphs represent the mean ± SEM of two independent biological replicates each measured in triplicate. ChIP was performed as described on HD11 cells 24 h after transfection

Our observations have several implications, some of which were addressed by chromatin immunoprecipitation (ChIP) experiments in HD-11 cells. We first asked whether the inability of c-Myb D152V to activate *mim*-*1* is caused by impaired binding to the *mim*-*1* enhancer and promoter chromatin regions compared to wild-type c-Myb. To address this question, we performed ChIP experiments after transfection of HD-11 cells with either wild-type c-Myb or the D152V mutant and monitored occupancy by qRT-PCR. The c-Myb responsive *mim*-*1* locus is composed of a promoter proximal region with two strong Myb recognition elements (MRE: TAACGG, see [27]) originally termed A and B in [19]. In addition, an upstream enhancer 2 kb upstream was identified [28], characterized by a cluster of MREs, as illustrated in Fig. 5a. As shown in Fig. 5b, c, both regions clearly bind wild-type c-Myb and the D152V mutant. In fact, the activation defective D152V apparently bound more efficiently than the wild-type c-Myb, which might be caused by its higher expression level (Figs. 3 and 4). Overall, this observation is consistent with our previous conclusion that the D152V mutant is impaired in pioneer function (chromatin opening), not in DNA binding [13]. The lost ability to activate the *mim*-*1* gene is therefore not caused by differential chromatin binding.

We next asked whether we by ChIP could observe changes in H3K27ac levels at the *mim*-*1* enhancer and promoter. We found that the D152V mutant caused only minor alterations in the acetylation levels compared to empty vector control, suggesting that it does not enable p300-mediated acetylation (Fig. 5e–g). The mutant appears to bind chromatin, but without inducing any major change in acetylation. In contrast, wild-type c-Myb induced a drop in the level of H3K27ac at both the enhancer and the promoter. At first, we thought this might be inconsistent with our model, until we realized that H3K27ac ChIP monitors the level of both H3 and its modification H3K27ac. If c-Myb in fact acts as a pioneer factor and through its binding displaces nucleosomes and creates a nucleosome-free region, this drop is exactly what we should expect. Moreover, from published ChIP-seq data we know that c-Myb, like many other TFs, is found in “valleys” of the acetylation profile (exemplified in [29], specifically its Fig. 5c). This is also the basis for DNaseI hypersensitive sites [30]. In our ChIP analysis we sonicated the chromatin down to a range of 100-300 bp (Fig. 5d), thus revealing only the closest neighbourhood of the bound factor. The acetylation drop observed with wild-type c-Myb, not with the D152V mutant, is therefore consistent with the pioneer model. Unfortunately, it does not directly address the role of acetylation in the transition from closed to open chromatin. To capture this transition, if at all possible, would have required a highly dynamic ChIP analysis using an inducible system with extremely fine time resolution.

We next asked how TSA treatment affected the acetylation levels at the *mim*-*1* locus. As shown in Fig. 5e–g, we observed the expected increase in H3K27ac levels at both the promoter and the enhancer after TSA treatment, independent of c-Myb. Again, the D152V mutant caused little change in this enhancement, while the wild-type c-Myb caused a reduced H3K27ac level, consistent with less nucleosomes at the c-Myb bound sites. Taking into consideration that the ChIP assay was performed using transfected cells, the remaining TSA-induced increase in a region occupied by wild-type c-Myb is probably related to the fact that TSA affects all cells, while c-Myb is present only in the transfected fraction of cells.

## Discussion

**Fig. 6:**
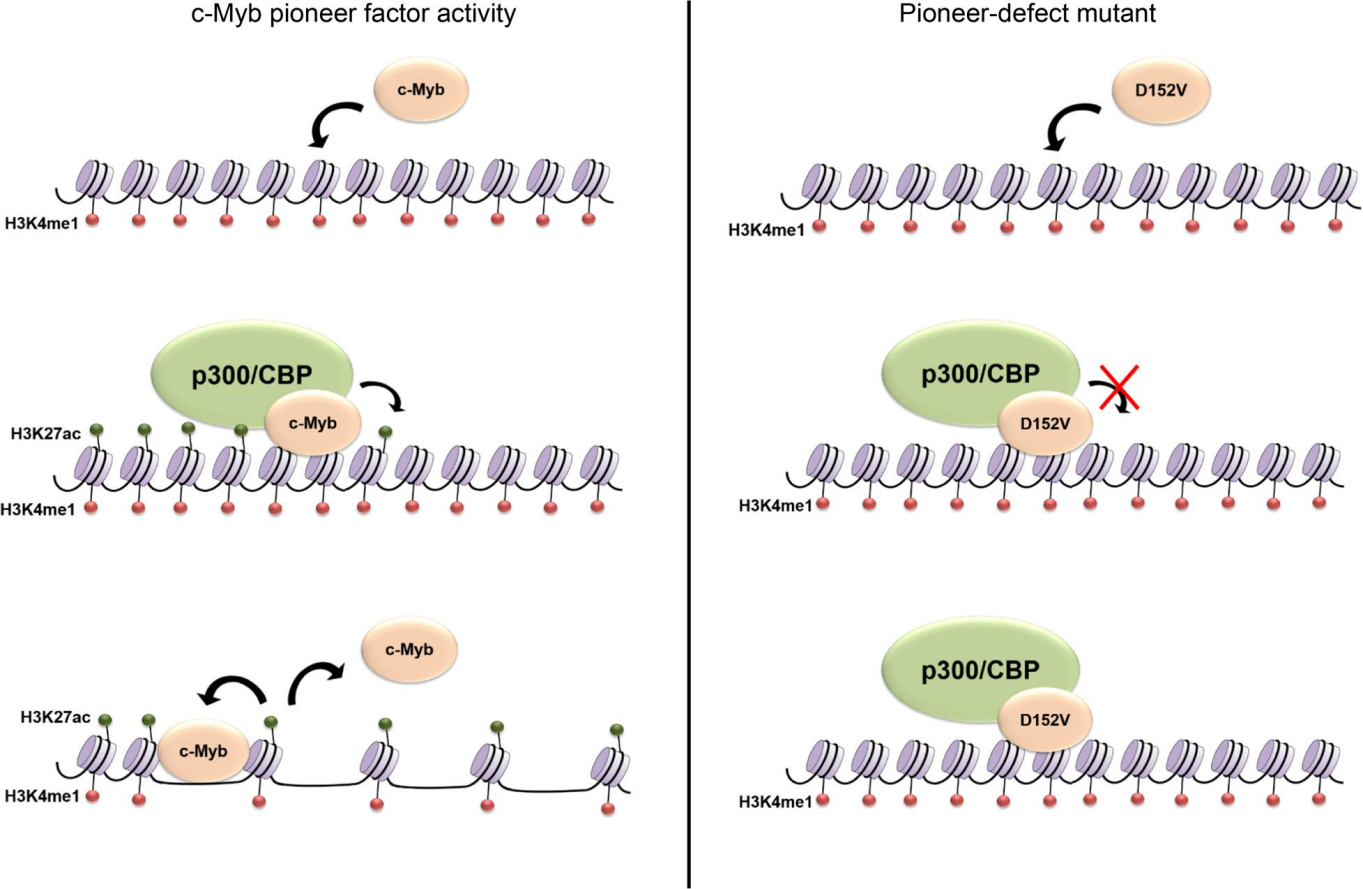
Pioneer factor model. We propose a model for the pioneer factor function of c-Myb (left panel) where c-Myb binds to closed chromatin, possibly marked by H3K4me1, then recruits H3K27 acetyltransferases such as p300/CBP, facilitating histone acetylation. Due to the reduced nucleosomal stability caused by histone acetylation, chromatin accessibility increases and c-Myb detaches from chromatin because of the lost interaction with acetylated histones. The pioneer-defect D152V mutant (right panel) binds to chromatin and recruits H3K27 acetyltransferases, but is unable to facilitate H3K27ac due to the weakened histone interaction. Therefore, the D152V mutant is unable to increase chromatin accessibility, and the chromatin-embedded target gene remains silent

In this study, we have identified mechanistic details of how c-Myb acts as a pioneer factor and activates expression of genes in closed chromatin. We show that to activate transcription of a chromatin-embedded gene, the interaction of c-Myb with histones and recruitment of p300 is essential and possibly involves induction of histone acetylation. Furthermore, we show that histone acetylation, and particularly H3K27ac, prevents c-Myb from binding to histones. The latter provides new insight into pioneer factor binding dynamics and the consequences of histone modifications. Based on our observations, we suggest a model for c-Myb pioneer factor activity where c-Myb first binds to closed chromatin and recruits p300/CBP here. p300/CBP then acetylates histones, which loosens the chromatin compaction and causes c-Myb to detach from nucleosomes (Fig. 6, left panel). However, after opening c-Myb can reengage in pure DNA binding at its recognition site in the nucleosome-free region. In this model, the pioneer-defect D152V mutant would bind to closed chromatin and recruit p300/CBP. However, due to the weakened histone interaction of the mutant, it may be unable to facilitate histone acetylation and therefore unable to increase chromatin accessibility and the chromatin-embedded target gene remains silent (Fig. 6, right panel).

Identifying a pioneer-defect mutant of c-Myb, D152V, provided us with a useful tool to study its pioneer factor function. This is the first pioneer-specific mutant of any transcription factor identified so far, where this particular function is impaired while keeping its DNA binding intact, and might help to dissect pioneer mechanisms in a way that will be relevant to pioneer factors in general. Interestingly, we found that the binding pattern of the D152V mutant to histone tails was identical to that of wild-type c-Myb, binding to all four canonical histones and only disrupted by specific modifications (Fig. 2). This suggests that the R2 repeat of the DBD of c-Myb is able to bind to histone tails in the same manner as the full DBD, as D152V is located to the R3 repeat and disrupts histone binding of this repeat only. The binding pattern of R3 alone was also identical to that of the full DBD, further suggesting that R2 and R3 bind to histone tails equally (Additional file 1: Figure S1). Even though the histone binding is lost in the R3 by D152V, R2 seems to be fully capable of binding to histones independently of R3. Due to the severely impaired ability of c-Myb D152V to open chromatin, the R3 repeat seems to be important for the chromatin opening step rather than the binding to closed chromatin [13].

Three H3 PTMs specifically disrupted the binding of c-Myb to the H3 tail, namely H3R26cit, H3K27ac and H3S28P (Fig. 2). Interestingly, other modifications of the same residues had no apparent effect on c-Myb binding. H3K27ac is the most studied of these three PTMs, and it is well known that c-Myb recruits the H3K27 acetyltransferase p300/CBP to target genes. So far, most studies are mainly focused on how acetylated histones act as binding platforms for bromodomain proteins or other acetylation readers, but to our knowledge no studies have observed the opposite; that histone acetylation may disturb the binding of other proteins. However, there is evidence that the combination of H3S10P and H3K14ac cause dissociation of HP1 proteins from H3 allowing the cell to overcome HP1-mediated transcriptional repression [31]. Since H3K27ac has such a specific effect on c-Myb binding, we hypothesize that c-Myb might detach from chromatin after having recruited H3K27 acetyltransferase activity to its target sites and that it is the histone modification that causes the chromatin to open rather than the pioneer factor itself. Likewise, the less studied modifications H3R26cit and H3S28P are also associated with open chromatin [17, 32]. To date, c-Myb is not known to recruit histone deiminases or histone kinases to its target genes.

Although c-Myb seems to bind several regions of H3, modifications in residue 26–28 have a very specific effect on its binding. Residue 27–42 of H3 has previously been suggested to be the binding site of c-Myb [23], and our findings may reflect a stronger binding to this region in the absence of the respective modifications. The histones are predominantly globular, except for their unstructured N-terminal tails. One possible reason for c-Myb to bind stronger to this region of H3 and be more sensitive to modifications here could be that it is closer to the globular domain of H3 and consequently closer to the region that is bound by DNA in a nucleosome. Since it is the DBD of c-Myb that binds both DNA and histones, nucleosome binding by c-Myb may require that the binding sites on histones and DNA are in close proximity. Histone modifications that decrease the positive charge of the histone tail will consequently cause a weaker interaction between the histone tail and DNA, as well as between the histone tail and the acidic patches in the c-Myb DBD that interacts with histones. One may imagine that c-Myb is not able to bind its DNA recognition motif on the nucleosome core without also binding the basic histone tails and that c-Myb will dissociate from chromatin when H3 is acetylated, phosphorylated and/or citrullinated at residue 26–28. This dissociation of factors from chromatin may be a process in which the cell controls the dynamics of transcriptional events, making sure that factors are evicted and possibly degraded after having exerted their function on chromatin.

The importance of the histone binding site in R3 to be intact for c-Myb to open chromatin might suggest that R3 is involved in facilitating specific histone modifications [13]. The DBD of c-Myb has previously been suggested to position H3 for acetylation by p300, leading to increased histone acetylation and gene activation [23]. It is therefore conceivable that R3 positions the H3 tail for its subsequent modification by histone modifying enzymes and that this function is impaired by the D152V mutation. TSA treatment, which enhances histone acetylation, was able to override the repression of *mim*-*1* in HD-11 cells transfected with D152V and even in cells without c-Myb expression, whereas the treatment had no effect on *mim*-*1* expression in cells transfected with wild-type c-Myb. Based on western blot signals, the TSA treatment enhanced global H3K27ac levels equally in all the cells (Fig. 3). The ChIP analysis also confirmed that the TSA treatment caused enhanced H3K27 acetylation at the c-Myb responsive regions at the *mim*-*1* locus (Fig. 5e–g). Enhancement of histone acetylation restored *mim*-*1* expression even in the absence of c-Myb. Considering the dependence of *mim*-*1* expression on c-Myb’s pioneer factor activity, the underlying mechanism of this pioneer function is likely to involve introduction or increase of histone acetylation in particular, and possibly other activating histone modifications as well.

Despite the obvious role of acetylation in our observations and model, there are some details pointing to a greater complexity. The ability of TSA to increase *mim*-*1* expression in the absence of c-Myb (Fig. 3) combined with its ability to increase the H3K27ac levels at the *mim*-*1* locus (Fig. 5e–g) suggests a slightly different activation mechanism in the two cases. While c-Myb-induced activation seems to imply loss of nucleosomes and bound c-Myb, TSA-induced activation appears to occur without full displacement of nucleosomes (reflected in enhanced H3K27ac levels) but with generation of acetylated nucleosomes, suggesting that some nucleosomal features, such as acetylation-induced changes in accessibility or nucleosomal architecture [33–35], are sufficient for *mim*-*1* activation.

The interaction between c-Myb and p300/CBP is required for normal haematopoiesis, but also crucial for the aberrant gene activation that occurs during leukaemic transformation due to c-Myb overexpression [24, 36]. It was therefore interesting to find that a c-Myb mutant, M303V, supposed to have a weakened p300 interaction [24], has the same defect in activating transcription of the *mim*-*1* gene as a mutant with a weakened histone interaction, namely D152V (Fig. 4). Mice harbouring either of these two mutations have been found to have very similar phenotypes, including several haematopoietic defects, suggesting that they do affect a similar mechanism [24, 25]. This suggests that in order for c-Myb to activate transcription of *mim*-*1*, interaction with both histones and involvement of p300 is essential, possibly because histone acetylation is needed for the activation and c-Myb might position H3 for acetylation by p300. Transcriptional activation of the *mim*-*1* gene has been found to involve chromatin opening by c-Myb [20, 21], as also our ChIP analysis indicates (Fig. 5), but it might in fact be the histone acetylation that is responsible for the increased chromatin accessibility due to reduced nucleosomal stability, either by itself or by somehow in cooperation with the pioneer factor [6]. By positioning histone tails for acetylation, c-Myb could be regarded as a cofactor for p300, allowing the latter to more efficiently acetylate sites in closed chromatin. The pioneer function of c-Myb would then be closely related to this p300 cofactor function. Of note, almost all pioneer factors identified so far interacts with p300/CBP [8, 37–44]. A few studies have also shown that pioneer factors that recruit p300/CBP to enhancers also facilitate H3K27ac at the enhancer, which might indicate that this is a general mechanism of action of pioneer factors [8–10]. Overexpression of p300 was able to partially override the defect of the two c-Myb mutants in HD-11 cells, in the same way as enhancement of histone acetylation was able to restore activation of *mim*-*1* in the absence of c-Myb (Figs. 3 and 4), suggesting that *mim*-*1* expression might not completely depend on c-Myb transcriptional activity.

## Conclusions

Most pioneer factors identified so far interact with chromatin modifiers, including p300/CBP. Whether pioneer factors are able to increase chromatin accessibility fully independent of other factors is still being debated [2, 45]. Based on the data presented here, we propose a mechanism of c-Myb pioneer factor function in which both recruitment of histone acetyltransferase activity and induction of histone acetylation is essential for the opening of chromatin and activation of gene expression. Since recruitment of p300 is a common feature of many transcription factors, we propose that the pioneer function of c-Myb is specifically dependent on its ability to bind histones, thereby acting as a cofactor for p300 needed to efficiently acetylate H3K27 at c-Myb bound sites in inaccessible chromatin. As the H3K27ac mark is commonly associated with active enhancers, this may represent a mechanism by which c-Myb promotes enhancer activation. We suggest that this mechanism may be applicable to other pioneer factors as well. In addition, we have revealed a previously unknown outcome of histone acetylation, namely restricting pioneer factor binding to chromatin.

## Methods

### Cell culture

Three cell lines were used in this work: K562 (ATCC^®^ CCL-243™*Homo sapiens* bone marrow, chronic myelogenous leukaemia), COS-1 (ATCC^®^ CRL-1650™ *Cercopithecus aethiops* kidney) and HD-11 (*Gallus gallus* macrophage). The HD-11 cell line has been described [46] and was kindly provided by A. Leutz (Max Delbrück Center for Molecular Medicine, Berlin). The cells were cultured as described in [47]. K562 cells were treated with 50 ng/ml TSA for 4 h at 37 °C before harvesting. HD-11 cells and COS-1 cells were seeded and transfected with plasmids using TransIT-LT transfection reagent (Mirus Bio) as described in [48]. HD-11 cells were treated with 15 ng/ml TSA for 16 h at 37 °C (8 h after transfection) before harvesting. RNA was isolated from HD-11 cells 24 h after transfection. RNA isolation, reverse transcription and qRT-PCR were performed as described in [13]. Protein extraction from HD-11 cells and western blotting was performed as described in [13]. COS-1 cells were lysed 24 h after transfection and used in co-immunoprecipitation assays.

### Purification of recombinant and native histones from K562 cells

Recombinant histones were expressed in *E. coli* BL21(DE3)pLysS, purified and reconstituted into octamers as described previously [49]. The protocol for purification of native histones is based on [50] and [51]. K562 cells were treated with 50 ng/ml TSA or DMSO for 4 h at 37 °C before harvesting and wash in ice-cold 1x PBS. Cells were resuspended in ice-cold NBA/B buffer (85 mM NaCl, 5.5% sucrose, 10 mM Tris–HCl pH 7.5, 0.2 mM EDTA, 0.05% NP40, 0.2 mM PMSF, 1 mM DTT, 1x Complete protease inhibitor cocktail (Roche)) and incubated for 3 min on ice. 5 mM sodium butyrate was added to all subsequent buffers for TSA-treated cells. Cells were centrifuged at 2500 rpm and resuspended again in NBA/B buffer, and the procedure above repeated. Nuclei were washed in ice-cold NBR buffer (85 mM NaCl, 5.5% sucrose, 10 mM Tris–HCl pH 7.5, 3 mM MgCl_2_, 1.5 mM CaCl_2_ 0.1% NP40, 0.2 mM PMSF, 1 mM DTT, 1x Complete protease inhibitor cocktail) and resuspended in 10 mL NBR buffer and subjected to MNase digestion with 214 Boehringer units for 10^8^ cells for 10 min at 37 °C with occasional swirling to obtain mononucleosomes. Reactions were stopped by adding EDTA to a final concentration of 30 mM and centrifuged for 10 min at 8000 rpm at 4 °C and resuspended in TE buffer containing 0.2 mM PMSF, 1 mM DTT and 1x Complete protease inhibitor cocktail. The reaction was centrifuged again at 12 000 rpm at 4 °C and salt concentration adjusted to 0.63 M NaCl and 100 mM K-PO_4_ pH 7.2. Octamers were loaded onto a prepacked hydroxyapatite column in 0.63 M NaCl and 100 mM K-PO_4_ pH 7.2 and eluted in high-salt buffer (2 M NaCl, 100 mM K-PO_4_ pH 7.2). Pooled fractions of octamers were concentrated on an Amicon ultra-15 centrifugal filter unit (cut-off 3 kDa, Merckmillipore) and stored in 50% glycerol at − 20 °C.

### GST pull-down

GST and GST fusion proteins were expressed in *E. coli* as previously described [52]. GST and GST fusion proteins prebound to Glutathione Sepharose beads (GE Healthcare) were incubated for 1 h rotating at 4 °C with 6 µg purified histone octamers from K562 cells in interaction buffer (20 mM HEPES pH 7.6, 10% glycerol, 0.2% Triton-X-100, 150 mM KAc, 1 mM DTT, 1x Complete protease inhibitor cocktail). Beads were washed 4 times in interaction buffer with 10 min rotation at 4 °C for each wash and resuspended in SDS loading buffer after the last washing step. Protein was detected by western blotting as described in [13].

### Peptide binding assays

MODified™ histone peptide arrays (13,005, Active Motif) were blocked in TBST buffer (10 mM Tris–HCl pH 7.5, 0.05% Tween 20, 150 mM NaCl) containing 5% non-fat dried milk at 4 °C overnight. The membranes were incubated with 10 nM GST-tagged c-Myb or GST protein alone at 4 °C overnight in interaction buffer (20 mM HEPES pH 7.6, 10% glycerol, 0.2% Triton-X-100, 150 mM KAc, 1 mM DTT, 1x Complete protease inhibitor cocktail). After washing in TBST, the membranes were incubated with mouse anti-GST (GenScript) in TBST (1:2000 dilution) containing 5% non-fat dried milk at 4 °C overnight. After washing in TBST, the membranes were incubated with anti-mouse IRDye 800CW (LiCor) in TBST (1:10 000 dilution) containing 5% non-fat dried milk for 1 h at room temperature and after washing in TBST, the membranes were imaged using the Odyssey imaging system. The arrays were analysed using the manufacturer’s software (Active Motif). A full overview of the peptide spot positions can be found here: https://www.activemotif.com/catalog/668/modified-histone-peptide-array.

### Co-immunoprecipitation

COS-1 cells were lysed 24 h after transfection in lysis buffer (20 mM HEPES pH 7.6, 10% glycerol, 1.5 mM MgCl_2_, 150 mM KAc, 0.05% NP-40, 1 mM DTT, 1 mM PMSF, 5 × Complete protease inhibitor cocktail). Lysates were subjected to immunoprecipitation with indicated antibodies and Protein A Dynabeads (Invitrogen) for 2 h rotating at 4 °C. The beads were washed 2 times in lysis buffer and resuspended in SDS loading buffer after the last washing step. Protein was detected by western blotting as described in [13].

### Chromatin immunoprecipitation

The HD-11 cell line was transfected with plasmids encoding TY-tagged c-Myb variants (empty vector, 3xTY1-c-Myb, 3xTY-c-Myb D152V) described in [13]. The ChIP analysis was performed essentially as previously described [53, 54], using anti-Ty1 antibodies for IP of c-Myb and anti-H3K27ac antibody (# 39,133, Active Motif) for IP of H3K27ac. The anti-Ty1 monoclonal mouse antibody was produced in our lab from a hybridoma cell line [55]. Occupancy was determined with qRT-PCR using primers for specific regions of the *mim*-*1* locus (*LECT2*). Primer sequences are available upon request.

### Chromatin extraction assay

Nuclei were isolated from K562 cells stably expressing TY-tagged c-Myb or c-Myb D152V. Salt extractions were performed essentially as described in [56]. Sedimented material (nuclear pellet P) and methanol/chloroform-precipitated supernatant (S) proteins were dissolved in SDS sample buffer and analysed as for Western blots.

### Plasmids

The mammalian expression vectors pCIneo-hcM-HA, pCIneo-hcM (D152V)-HA, pCIneo-hcM (M303V)-HA, pCIneo-hcM (D152V-M303V), pCMV-p300-Myc as well as “empty vector” pCIneo-HA were used for expression of c-Myb and p300 in HD-11 cells and COS-1 cells. pCIneo-hcM-HA, pCIneo-hcM (D152V)-HA and pCMV-p300-Myc have been previously described in [13, 53, 57]. The M303V mutation was generated using PCR-based mutagenesis on a subfragment of human c-Myb, and the D152V-M303V double mutation was generated by combining restriction fragments (using EcoRI + NotI) from the single mutation plasmids.

The expression vectors for the GST fusion proteins GST-hcM-NR123, GST-hcM-NR123 (D152V), GST-hcM-R3 and GST-hcM-R3 (D152V) have been described in [13].

## Additional files

**Additional File 1: Figure S1**. Peptide arrays containing 384 histone tail modification combinations incubated with GST-c-Myb-R3 (left) or GST-c-Myb-R3-D152V (right) and detected with anti-GST primary antibody.

**Additional File 2: Figure S2**. The same experiment as shown in Fig. 3, but here RNA was analysed for expression of *lysozyme* (*LYZ, Gallus gallus (Chicken))* measured by qRT-PCR. The values of RNA expression were normalized to the relative amount of the reference gene *hprt*.

**Additional File 3: Figure S3**. Isolated nuclei were extracted with 0.1% TX-100 and increasing NaCl concentrations, and soluble (S) and insoluble (P) nuclear fractions analysed by western blotting using anti-Ty antibodies to visualize c-Myb and c-Myb D152V. Densitometric analysis of salt extractions are shown in upper row for c-Myb (middle row) and c-Myb D152V (bottom row).
