# Supplementary figures and images for: The pioneer factor activity of c-Myb involves recruitment of p300 and induction of histone acetylation followed by acetylation-induced chromatin dissociation
Source: Epigenetics Chromatin. 2018 Jun 28;11:35. doi: 10.1186/s13072-018-0208-y (PMC6022509; doi:10.1186/s13072-018-0208-y)

**Figure S1**

**GST-c-Myb-R3**

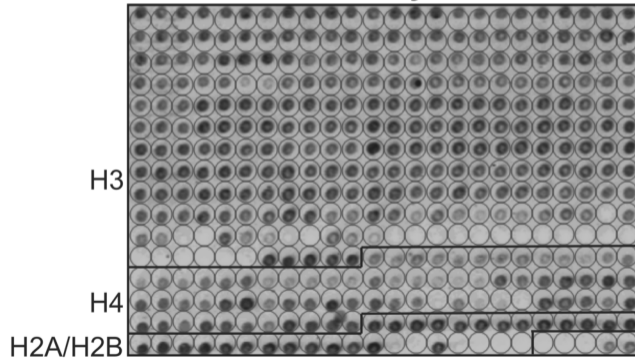

**GST-c-Myb-R3-D152V**

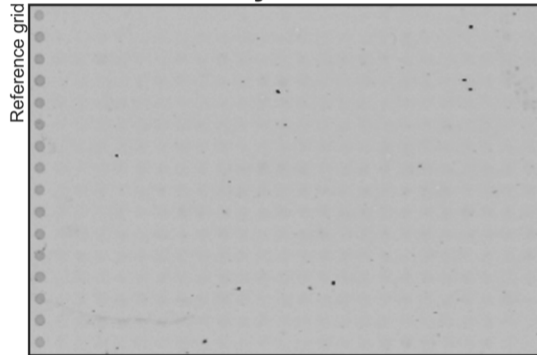

Supplement: Supplementary file 1 — Additional File 1: Figure S1. Peptide arrays containing 384 histone tail modification combinations incubated with GST-c-Myb-R3 (left) or GST-c-Myb-R3-D152V (right) and detected with anti-GST primary antibody. [file 13072_2018_208_MOESM1_ESM.pdf]

# Figure S2

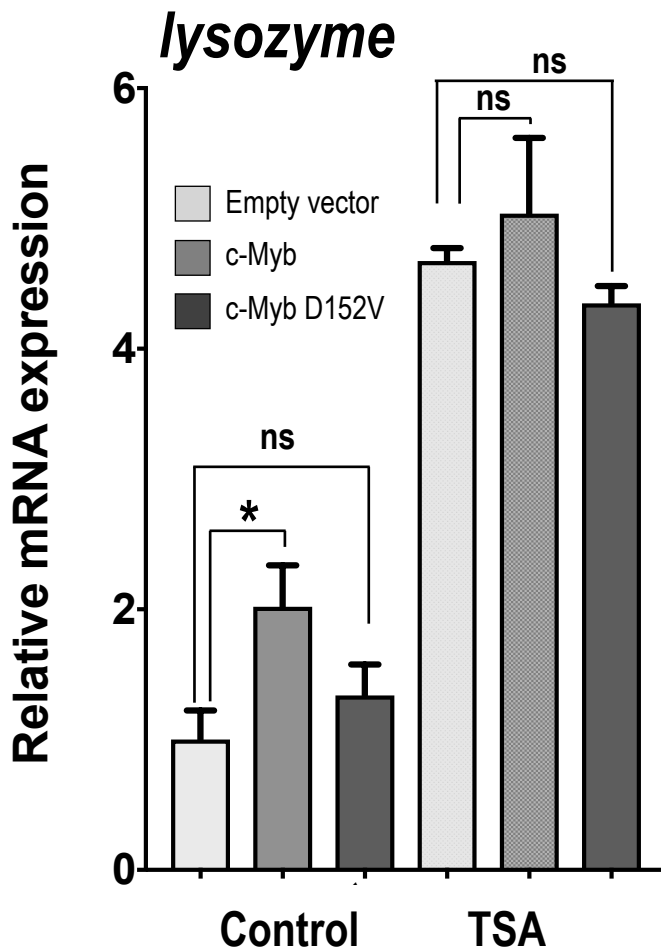

Supplement: Supplementary file 2 — Additional File 2: Figure S2. The same experiment as shown in Fig. 3, but here RNA was analysed for expression of lysozyme (LYZ, Gallus gallus (Chicken)) measured by qRT-PCR. The values of RNA expression were normalized to the relative amount of the reference gene hprt. [file 13072_2018_208_MOESM2_ESM.pdf]

# Figure S3

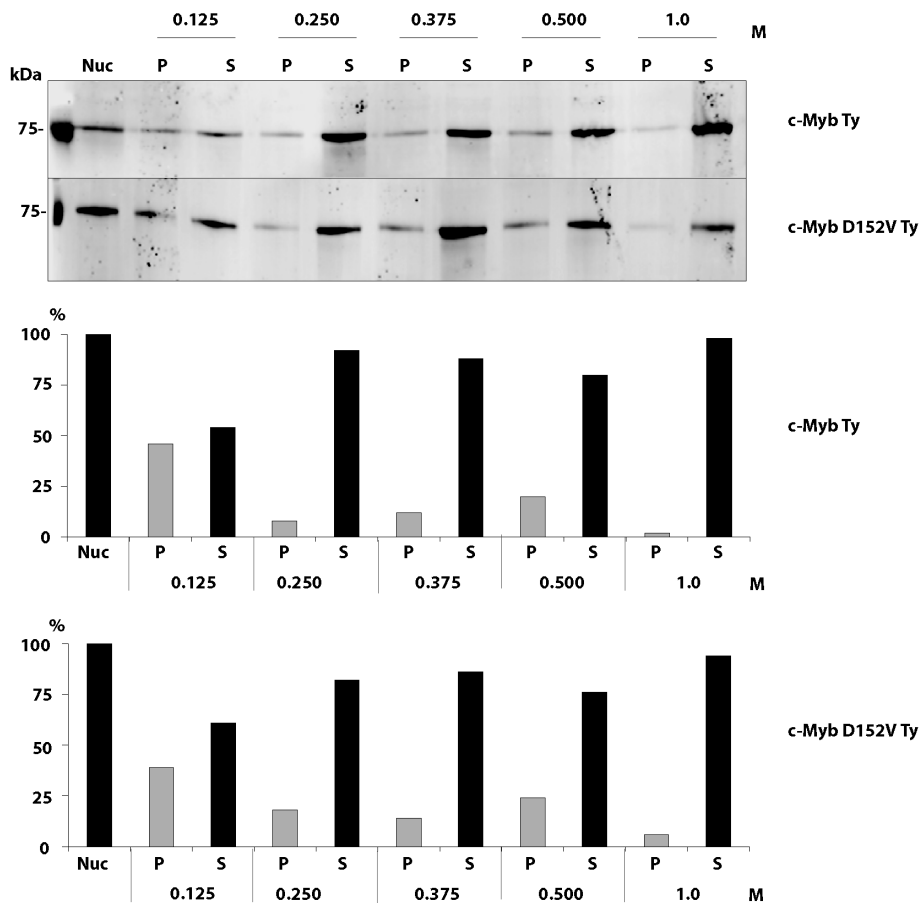

Supplement: Supplementary file 3 — Additional File 3: Figure S3. Isolated nuclei were extracted with 0.1% TX-100 and increasing NaCl concentrations, and soluble (S) and insoluble (P) nuclear fractions analysed by western blotting using anti-Ty antibodies to visualize c-Myb and c-Myb D152V. Densitometric analysis of salt extractions are shown in upper row for c-Myb (middle row) and c-Myb D152V (bottom row). [file 13072_2018_208_MOESM3_ESM.pdf]
